# Microfluidic devices for precise measurements of cell directionality reveal a role for glutamine during cell migration

**DOI:** 10.1038/s41598-023-49866-9

**Published:** 2023-12-27

**Authors:** Nil Gural, Daniel Irimia

**Affiliations:** 1 Center for Engineering in Medicine and Surgery, Department of Surgery, Massachusetts General Hospital, Boston, MA USA; 2 Harvard Medical School, Boston, MA USA; 3 Shriners Hospitals for Children, Boston, MA USA

**Keywords:** Engineering, Microfluidics

## Abstract

Cancer cells that migrate from tumors into surrounding tissues are responsible for cancer dissemination through the body. Microfluidic devices have been instrumental in discovering unexpected features of cancer cell migration, including the migration in self-generated gradients and the contributions of cell–cell contact during collective migration. Here, we design microfluidic channels with five successive bifurcations to characterize the directionality of cancer cell migration with high precision. We uncover an unexpected role for glutamine in epithelial cancer cell orientation, which could be replaced by alfa-keto glutarate but not glucose.

## Introduction

Cancer cell migration and cancer cell metabolism are two hallmarks of cancer. While cancer cell migration is responsible for the invasion and dissemination of cancer cells^1^, cancer cell metabolism is vital for cancer cell proliferation and tumor growth^2^. Together, the invasive and growth processes are responsible for forming metastases that cause death in 90% of cancer patients. However, despite their importance, the metabolism and migration of cancer cells are most often studied independently. It is commonly assumed that cells can produce the energy needed for migration by using various substrates^3,4^, and, as such, cell migration is unlikely to be affected by the energy source in the cell.

Among the various metabolic substrates employed by cancer cells, glutamine is the focus of intense research and is regarded as a potential target in anti-cancer treatments ^5^. However, the role of glutamine in cell migration remains controversial. Some recent studies suggest that cell migration requires glutamine^6,7^, while others suggest that the lack of glutamine enhances cancer cell migration^8^. One significant challenge in probing the relationship between glutamine metabolism and cell migration is the lack of specificity of the traditional cell migration assays^9^. The assays commonly employed for testing cell migration are also sensitive to changes in cell proliferation^10^. While cell proliferation is also dependent on glutamine, one cannot distinguish, using current assays, the effect of glutamine on cell migration from the effect of glutamine on proliferation. Isolating the relationship between glutamine metabolism and cell migration from potential confounding factors requires new, specialized assays.

Our understanding of cancer cell migration has benefited significantly in the past decade from the emergence of microfluidic technologies. Microfluidic tools helped elucidate the contribution of self-generated gradients of epidermal growth factor (EGF) in cancer cell orientation during cancer migration^11^ and probe the molecular signaling pathways during the epithelial-to-mesenchymal transition^12^. The range of microfluidics tools available today for probing cell migration is expanding continuously, and today includes tools for precision measurements of cell migration in conditions that replicate in vivo mechanical confinement^13^, the isolation of rare cancer cells from circulation^14^, and the in vitro replication of immune-cancer cell interactions^15^.

Here, we design microfluidic devices with a series of bifurcating channels to study the directionality of cancer cells during the migration in self-generated EGF gradients. We probe the effect of glutamine on the orientation of cancer and non-cancer epithelial cells during migration. While the migration of cancer cells through channels is related to their in vivo metastatic potential, our results could have implications for uncovering new strategies aimed at reducing cancer invasion by interfering with the orientation of cancer cells during migration.

## Materials and methods

### Microfluidic devices

Microfluidic devices were fabricated using standard microfabrication techniques. Briefly, a 2 mm thick layer of polydimethyl siloxane (PDMS, Dow Corning, Midland, MA) was cast on a silicon wafer with photolithographic features. The silicon wafer supports three photolithographic layers. The first, 7 µm thick layer, defines the bifurcating channels through which the cells migrate. A second, 12 µm-thick layer defines a side channel inside which cells line up before moving into the bifurcating channels. The side channel is designed to maintain low cell density per unit surface and to line up the cells at the entrance of the channels at the beginning of each experiment. A third, 50 µm thick layer defines the loading channels and the connections to the outer compartments of the device. A central well is cut with a 0.75 mm hole punch, connecting the loading channels and the inlet port. Finally, each device is cut using a 5 mm hole punch. Forty-eight devices are exposed to oxygen plasma for 20 s (March, Concord, MA) and manually placed, in pairs, on a 24-well glass-bottom plate (Mattek) and baked for 6 min at 70 °C.

Immediately after bonding, 2 µL of 30 µg mL^−1^ collagen IV solution (Sigma Aldrich, St. Louis, MO) in Phosphate Buffer (PBS, Life Technologies, Grand Island, NY) is pipetted onto the center well, priming the devices. After a few minutes, when the channels are coated with collagen, each well is filled with approximately 1 mL of media with corresponding epithelial growth factor (EGF, Lonza Walkersville, Hopkinton, MA), glutamine (Sigma Aldrich, St. Louis, MO), and Dimethyl 2-oxoglutarate (AKG, Sigma Aldrich, St. Louis, MO) concentrations. The entire plate is then placed under a vacuum for 5 min to facilitate the removal of bubbles from the central well. Immediately after the vacuum treatment, the media in each well of the plate is removed, allowing only the central side channels and bifurcating channels to be coated with the media. For the directionality experiments, the microfluidic devices are filled with DMEM glucose and glutamine-free media supplemented with glutamine, glucose, or both, in addition to the required supplements.

### Cell preparation

The Non-Small Lung Adenocarcinoma Cell Line (PC9ZD expressing GFP–tubulin, a gift from Dr. Sri Sharma at the MGH Cancer Center) is cultured in RPMI media (Lonza Walkersville, Hopkinton, MA), supplemented with 10% Fetal Bovine Serum (FBS, Life Technologies, Grand Island, NY), 1% Penicillin/Streptomycin (Life Technologies, Grand Island, NY), 2 mM Glutamine and 1 mM Sodium Pyruvate (Sigma Aldrich, St. Louis, MO) in a humidified atmosphere of 5% CO2 in the air. Human mammary epithelial cells (HMEC, Lonza Walkersville, Hopkinton, MA) are cultured in Mammary Epithelial Growth Media (MEGM, Lonza Walkersville, Hopkinton, MA) supplemented with bovine pituitary extract, EGF, hydrocortisone, insulin and gentamicin/amphotericin-B (Lonza Walkersville, Hopkinton, MA), following standard protocols.

Prior to each experiment, the PC9 cells grown in culture flasks are rinsed with 1X PBS, and detached from the flask surface by incubation with 1.5 mL Accutase (Sigma Aldrich, St. Louis, MO) for 4 min. The cell release solution is then neutralized by adding 3.5 mL growth media. HMEC cells are washed with 5 ml of HEPES Buffered Saline Solution (HEPES-BSS, Lonza Walkersville, Hopkinton, MA) and detached from the surface by the addition of 2 mL Trypsin/EDTA (HEPES-BSS, Lonza Walkersville, Hopkinton, MA) and incubation for 2–6 min. After incubation, the cell release solution is neutralized by the addition of 4 mL of Trypsin Neutralizing Solution (TNS, Lonza Walkersville, Hopkinton, MA).

The cell suspension is then separated into tubes filled with growth media. The tubes are centrifuged at 1000 rpm for 5 min at 25 °C. After centrifugation, the cells are re-suspended at a concentration of ~ 1 million per milliliter in assay media formulations for each experiment. Suspensions of cells are loaded into each collagen-coated device by placing a 2–3 µL droplet of cells onto the central well. The plate is observed under a microscope until the side channels are filled with cells. Finally, 1–1.5 mL of assay media is added to each well to fully submerge the devices in the media.

### Assay media

Assay media is prepared by adding 1 ng/mL EGF and 1 mM sodium pyruvate into DMEM supplemented with 10% dialyzed FBS (Life Technologies, Grand Island, NY) and 1% penicillin/streptomycin. Different DMEM formulations with varying concentrations of glutamine and glucose are employed for the experiments (catalog numbers 11965 (glutamine, glucose), 11,054 (no glutamine, low glucose), 11,966 (glutamine, no glucose), and A1443001 (no glutamine, no glucose)—Thermo Fisher). For some studies, 2 mM AKG is added to the media.

### ATP measurements

Luminescence ATP Detection Assay System (ATPlite, Perkin Elmer, Waltham, MA) is used to monitor the ATP usage of cells in different media conditions. Cells are plated in a white, opaque 96-well plate (Corning, Tewksbury, MA) and left in 100 µL of their corresponding media for 1 h. Then, the assay is performed according to the manufacturer's recommendations.

### Image analysis

Time-lapse imaging is performed with a fully automated Nikon Ti-E microscope fitted with the perfect focus system and an environmental chamber maintained at 37 °C. Images are captured at 8 locations for each device every 20 min for 30 h. Cells move through the channels from a central compartment with cells towards the outer compartment without cells. At each bifurcation, cells could turn towards the dead-end or the through path. In addition, cells can turn back towards the cell compartment. To quantify the directional decisions, we count the cells that chose the through path at each of the five bifurcations. We normalize their number to the total number of cells that enter the channels.

### Statistical analysis

Each directional choice made by moving cells through the channels in the device is categorized as a binary choice, either through or dead-end. Up to five choices are quantified for the cells entering the channels with five bifurcations. Cells that made dead-end choices are not followed further even if they turn back and choose a through path. Only the first cells to enter each channel and reach at least one bifurcation are evaluated. Whenever a cell inside the channel undergoes division, it is excluded from the count.

To calculate an orientation factor (*b*), we start by counting the “through decisions” made by cells passing through each of the five bifurcations. We then normalize these counts to the number of cells entering the channels. Thus, from the experimental data, we calculate five fractions for “through decisions” for each bifurcation. In parallel, we calculate the proportion of cells that would take the “through path” at each of the five bifurcations relative to the number of cells entering the channels with the assumption that the orientation is uniform at all five bifurcations along the channel (a power series of *b*). We then calculate the orientation factors between 0.5 (random decisions) and 1 (always through the channel) for which the squared differences between the model and experimental measurements at the five bifurcations reach a minimum.

We compare the orientation factors for different experimental conditions using t-student tests or ANOVA statistics where appropriate. We only used experimental conditions for which more than 20 cells were observed entering the migration channels. We consider differences significant for *p* < 0.05.

## Results

**Figure 1:**
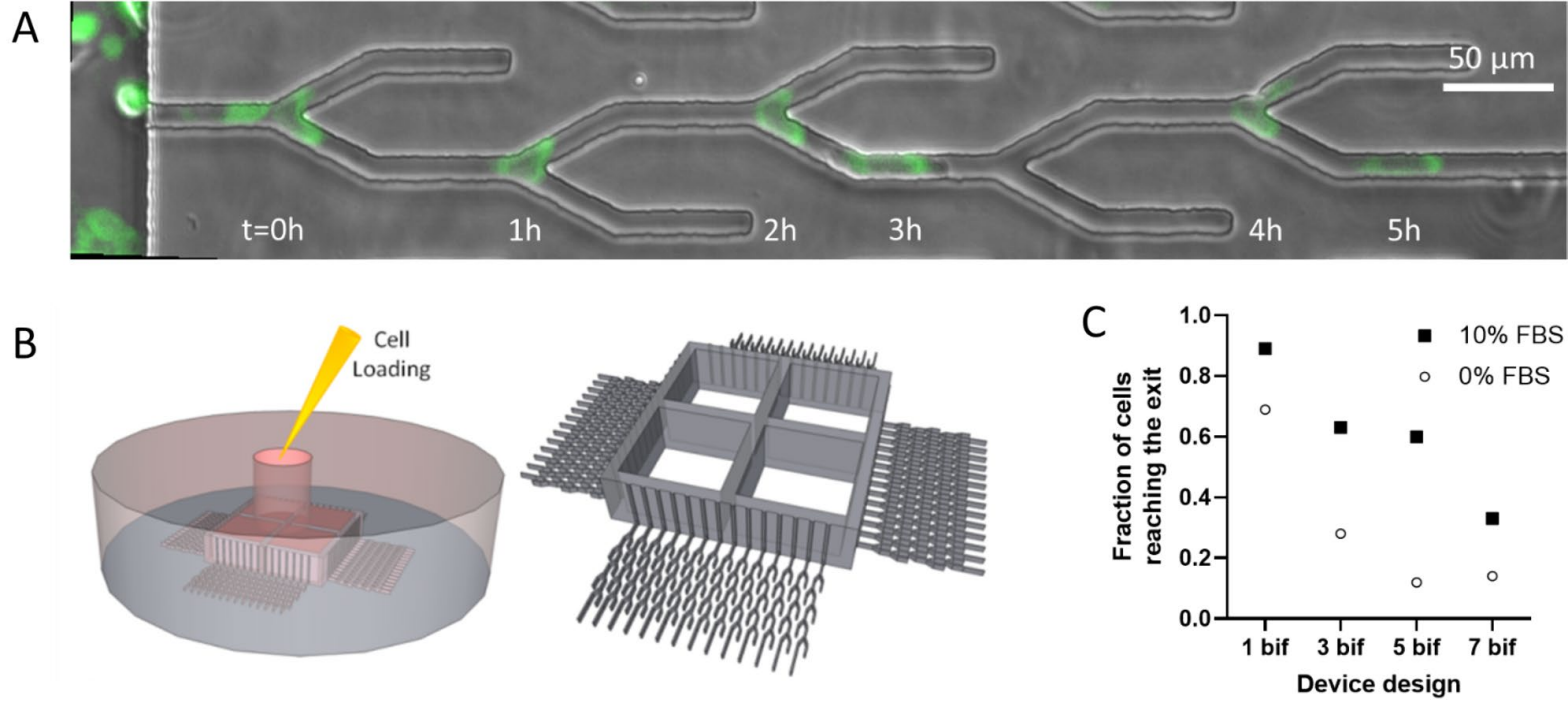
Microfluidic assay for precision measurements of cancer cell directionality during migration. (**A**) The location of a GFP-expressing PC9 cell at different times along one channel with five bifurcations is captured in overlapping images. In this example, the cell avoids the dead ends and exits the channel at 5 h. Scale bar is 50 µm. (**B**) Cells are loaded through a middle compartment. Cells sediment along the side channels, within 200 µm of the entrance to the bifurcating channels. (**C**) We calculated the fraction of cells that pass through channels with 1–7 successive bifurcations relative to the total number of first cells entering the migration channels. We compared the fractions in the presence and absence of serum in the media for N = 266, 33, 67, 27 cells entering the migration channels in a minimum of 3 independent devices with 1, 3, 5, 7 bifurcations in the presence of serum—filled squares; N = 228, 43, 31, 14 cells in a minimum of 3 independent devices with 1, 3, 5, 7 bifurcations in the absence of serum—open circles).

We designed microfluidic devices as tools to enable high-precision analysis of the directional choices made by moving cancer cells (Fig. 1, Supplementary Fig. 1). Inside these devices, cells are loaded as a cell suspension through a small inlet. The cell suspension then travels through the loading channels. The height of the channels has been empirically optimized to increase the density of cells settling from the suspension on the glass surface of the loading channel. During loading, the first cells settle at the entrance of the migration channels, aided by the cell alignment features. While the cross-section of the migration channels is smaller than that of the suspended cells, the settled cells are trapped at the entrance to the migration channels, inside the alignment features. Cell trapping at the entrance to migration channels restricts the fluid flow through those migration channels and prevents the crowding of the cells at the entrance to each channel. The combination of features ultimately helps distribute the cells uniformly through the device at the entrance of the migration channels.

After adhering to the bottom of the device, the epithelial cells start moving through the side channels and, through these, to an external reservoir of fresh media (Fig. 1A). The moving cells are confined in channels and pass through successive bifurcations where they could enter either dead-end or through channels (Supplementary Movie). The migration through channels is driven by an EGF gradient that self-establishes along the migration channel, driven by the competition between the EGF uptake by the cells in the loading channel and the EGF diffusion from the outer chamber. The self-generation mechanism for EGF gradients in this type of microfluidic device has been described in detail^11^.

We compared the yield of cells passing through channels with one, three, five, and seven bifurcations. We reasoned that by increasing the number of bifurcations, we could increase the number of repeated observations in similar conditions for each cell migrating along the channel. With the assumption that the orientation of cells is similar at different bifurcations, the increased number of observations increases the precision of evaluating each cell's performance. However, two factors limited the number of bifurcations in our assay. First, the number of cells reaching the end of channels decreases with the increasing number of bifurcations. Thus, the added benefit of increasing the number of bifurcations decreases with an increased number of bifurcations. Second, to facilitate the comparison of cell migration through channels with different numbers of bifurcations, the total length of the migration channel is similar in different devices with different numbers of bifurcations. We limited the number of bifurcations to 7 to avoid unnecessary long experiments. We then compared the directional decisions in channels with 1, 3, 5, and 7 bifurcations along migration channels of similar length. We observed a significant drop in the cellular yield in devices with 5 and 7 bifurcations per channel, with more cells ending trapped inside devices than going through (Fig. 1C). Thus, we chose for this study to use devices with 600 µm long channels, each with five bifurcations, which most cells traverse in approximately 8 h.

**Figure 2:**
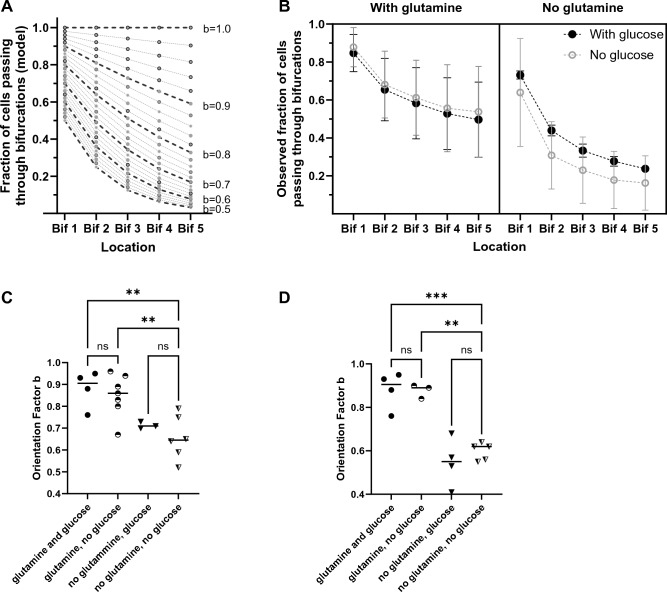
Fraction of cells moving through channels with bifurcations. (**A**) Calculated fractions of cells passing through each bifurcation of a five-bifurcations channel, assuming constant 'orientation factor' throughout the channel. Calculated values for orientation factors between 0.5 (random decisions) and 1 (always through the channel) are represented in 0.02 increments. (**B**) Experimental results show the fraction of PC9 cells passing through each bifurcation in the presence (left panel) and absence of glutamine (right panel). For each condition, the navigation of cells in the presence (filled black circles) and absence of glucose (empty gray circles) are presented in the same panel. We analyzed N = 767 (glutamine and glucose present), 622 (glutamine, no glucose), 312 (no glutamine, glucose), 351 (no glutamine, no glucose) cells that entered the migration channels; N = 7, 4, 3, 5 independent experiments, respectively). The dotted lines are a guide for the eye. (**C**) Glutamine emerges as the key factor in the differences in the orientation of PC9 cells, with significant differences between conditions with and without glutamine. One symbol is one experiment. The significance of differences was evaluated using ANOVA, * *p* < 0.05, ** *p* < 0.001. (**D**) The absence of glutamine, but not the absence of glucose, reduces the orientation of moving HMEC cells. One symbol is one experiment. The significance of differences was evaluated using ANOVA.

We quantified the bias of the cells moving through successive bifurcations using an *orientation factor b*. We defined the orientation factor as the bias towards the through channel when a cell arrives at a bifurcation. A value of the orientation factor b = 1 would indicate cells that are biased towards the through channel, b = 0.5 cells that make random decisions, and values below 0.5 cells that have a bias towards the dead-end channels all the time. Two key assumptions when calculating the orientation factor *b* are 1. that cells make independent decisions at each bifurcation and 2. that the probability of taking the through path does not change along the channel. Based on the definition and assumption, we then calculated, for uniform populations of ideal cells, the fraction of cells passing through at each of the five bifurcations relative to the cells entering the migration channels for orientation factors b with values between 1 and 0.5 (Fig. 2A). We then used these calculations and the measured frequencies for populations passing through the channels with five bifurcations to estimate *the orientation factor b*.

We employed the calculated orientation factor to compare the performance of cells in different conditions. We found that PC9 cells in growth media migrate robustly through the migration channels and display a strong bias towards the through channels. Out of 676 cells moving in the presence of growth media (with glutamine, glucose, and 10%FBS) through devices with five successive bifurcations, 409 reached the end, representing 58% of the cells. If cells were to make random choices at each bifurcation (b = 0.5), we would have expected no more than 3.1% of cells entering the migration channels to go through the entire channel. The twenty-plus-fold difference in the fraction of cells exiting the channel confirmed that a significant bias towards the through channels exists for PC9 cells in media. Our calculated orientation factor for the PC9 cells in growth media was b = 0.89 ± 0.03 (N = 6 independent experiments).

We then compared the cells moving through the channels with five bifurcations in the presence and absence of glucose and glutamine. To accurately control the concentration of glutamine and glucose during cell migration, we replaced the regular FBS with dialyzed FBS (10%). To this controlled media, we added glucose (5.5 mM), glutamine (2.0 mM), or both. We found that out of 767 cells that entered the channels in the presence of control media with glucose and glutamine, 398 went through all five bifurcations (b = 0.84 ± 0.08, N = 4). The orientation of the cells was statistically indistinguishable between control and growth media conditions (p > 0.05, t Student test, Supplemental Fig. 2).

When glutamine is absent from the media and glucose is present, the orientation decreases compared to the control media. Only 62 of 312 cells go through (b = 0.71 ± 0.02, N = 3, Fig. 2B) in the absence of glutamine. In the absence of glucose, the fraction of cells to go through is comparable to that in the controlled media (338 out of 622, b = 0.84 ± 0.12, N = 7). When both glucose and glutamine are absent from media, the orientation abilities of the cells moving through the bifurcations appear closer to that of cells in the absence of glutamine than in the absence of glucose (81 out of 348 cells, b = 0.65 ± 0.1, N = 6). Statistical analysis using two-way ANOVA confirms a significant effect of glutamine on orientation (**p < 0.005), the lack of effect of glucose on cell orientation (*p* > 0.05), and the absence of interaction between glutamine and glucose during cell orientation (*p* > 0.05, Fig. 2C).

To determine if the effect of glutamine on orientation is also true in non-cancerous epithelial cells, we analyzed the effect of glucose and glutamine on migrating HMEC cells (Fig. 2D). We calculated that the high orientation factor (b = 0.88 ± 0.08, N = 249 cells, N = 4 experiments) in control conditions decreases in the absence of glutamine (b = 0.62 ± 0.2, N = 210 cells, N = 4 experiments) but does not decrease in the absence of glucose (b = 0.88 ± 0.03, N = 95 cells, N = 3 experiments). When both glucose and glutamine are absent, an even smaller number of cells migrate, and none of them reaches the end of the channel (b = 0.6 ± 0.04, N = 243 cells, N = 5 experiments). Statistical analysis using two-way ANOVA confirmed a significant effect of glutamine on cell orientation (***p < 0.001), the lack of effect of glucose (*p* > 0.05), and the absence of interaction between glutamine and glucose during cell orientation (*p* > 0.05).

**Figure 3:**
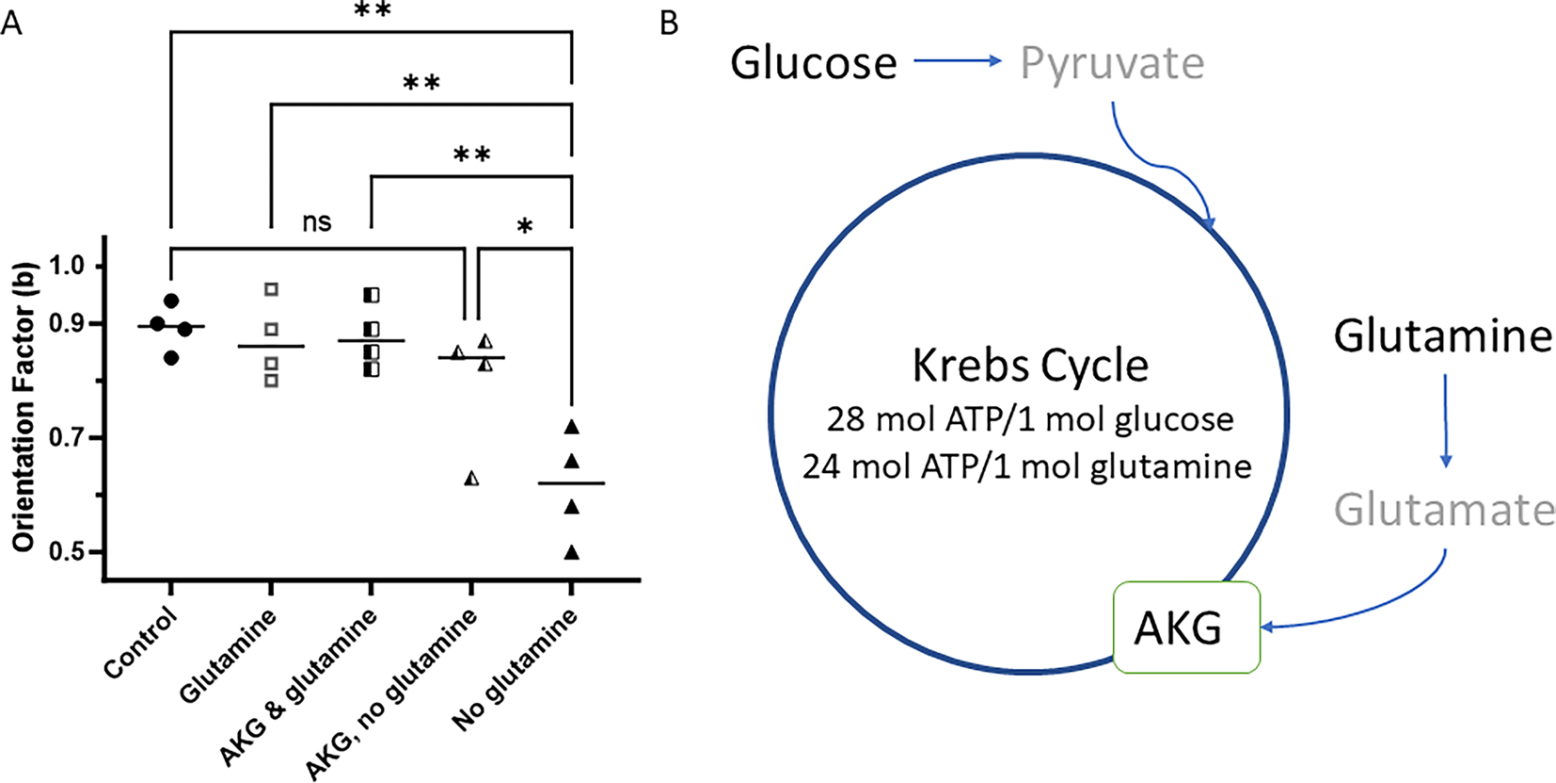
Alpha-ketoglutarate restores PC9 cell orientation at bifurcations. (**A**) The orientation of PC9 cells navigating through bifurcating channels, which is decreased in the absence of glutamine, can be restored by the addition of alpha-ketoglutarate. Control media with glucose and glutamine (full circles, N = 767 cells), glutamine and no glucose (empty squares, N = 622 cells), alpha-ketoglutarate and glutamine (half-filled squares, N = 337 cells), alpha-ketoglutarate and no glutamine (half-filled triangles, N = 279 cells), and no glutamine (full triangles, N = 348 cells). N = 4, 6, 4, 4, 6 independent experiments, respectively. The significance of differences was evaluated using ANOVA, * *p* < 0.05, ** *p* < 0.001. (**B**) A simplified schematic of the Krebs cycle showing the biochemical roles of glucose, glutamine, and AKG during the synthesis of ATP inside cells.

To start exploring the potential mechanisms by which glutamine contributes to cell orientation, we asked if the participation of glutamine in the Krebs cycle (tricarboxylic acid cycle) is important during cell orientation. Specifically, we first tested if alpha-ketoglutarate (AKG), an intermediate to the Krebs cycle derived from glutamine, could restore PC9 cell orientation in the absence of glutamine. We reasoned that AKG could enter the Krebs cycle as an alternative to glutamine. We found that the low orientation factor in the absence of glutamine (b = 0.65 ± 0.1, N = 6) can be significantly improved by the addition of AKG (b = 0.79 ± 0.11, *p* < 0.05, N = 4, Fig. 3). The PC9 cells in the presence of AKG and glutamine displayed comparable orientation (b = 0.88 ± 0.06, *p* > 0.05, N = 4 experiments) to cells in the presence of glutamine alone. These results suggest an interchangeable role of glutamine and AKG in PC9 cell orientation.

**Figure 4:**
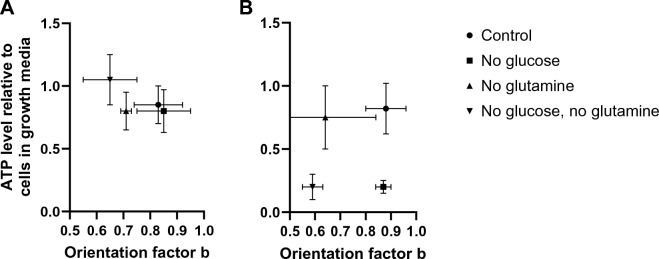
Orientation factor and cellular ATP levels. (**A**) ATP levels measured in PC9 cells are comparable for all media formulations despite differences in orientation factors (N = 767, 312, 622, 348 cells migrating through channels monitored for control, no glutamine, no glucose, and no glutamine, no glucose conditions; N = 4, 3, 7, 6 experiments, respectively). (**B**) ATP levels measured in HMEC cells decrease in the absence of glucose but not in the absence of glutamine, whereas the orientation factor is decreased in the absence of glutamine (N = 249, 210, 95, 243 cells migrating through channels monitored for control, no glutamine, no glucose, and no glutamine, no glucose conditions; N = 4, 4, 3, 5 experiments, respectively). Bars represent standard deviations.

While it is commonly assumed that glucose and glutamine metabolized through the Krebs cycle contribute to the energy needed for migration, we hypothesized that the absence of glutamine may reduce the energy available inside the cells and, through this, alter orientation. Thus, we measured the ATP levels for PC9 and HMEC cells in the presence and absence of glucose and glutamine in the media. We found no significant differences in the ATP levels in PC9 cells in the absence of glutamine in PC9 cells (Fig. 4A). We found no correlation between the ATP levels and the changes in PC9 orientation (reject the hypothesis of regression slope significantly non-zero, *p* > 0.05). Interestingly, we found that the ATP levels in HMEC cells do not change when glutamine is removed (Fig. 4B). We found no correlation between the ATP levels and the orientation changes (reject the hypothesis of regression slope significantly non-zero, *p* > 0.05). Interestingly, we determined that the ATP levels in HMEC cells decrease in the absence of glucose, even though removing glucose does not affect orientation. Overall, it appears that although glutamine is important for the orientation of both PC9 and HMEC cells during migration through channels, the effect may depend on the participation of glutamine in the Krebs cycle even when there are no significant changes in ATP levels in these cells.

## Discussion

We designed microfluidic devices to measure the orientation of epithelial cells during migration. Using these devices, we challenged the moving cells repeatedly at multiple bifurcations of identical geometry. We also developed a computational model to summarize the observations of individual decisions at each bifurcation into one *orientation factor*. We combined the microfluidic and computational tools and uncovered that glutamine plays a key role in epithelial cell orientation.

Innovative design elements of the microfluidic device include the use of multiple bifurcations in series. Unlike the traditional methods for evaluating cell orientation that rely on observations of cells on flat surfaces, when cells can turn at any angle in a 360-degree space, the microfluidic method relies on binary decisions (through vs. dead-end). The fundamental features of directional decisions are the same, where orientation results from random decisions and bias^16^. While binary decisions are clearer and easier to quantify, the precision of the orientation measurements is higher and allows for better comparisons between conditions. Small differences in orientation could be captured with accuracy. The use of multiple bifurcations in series enables finer resolution of the digitization process, whereas the possible outcome of directional decisions increases exponentially. In the case of five bifurcations, the number of outcomes increases from two to 32. Overall, this feature increases the amount of information available from experiments and, in conjunction with a computation model, helps make robust orientation measurements.

Additional design innovations include the high aspect ratio loading channels with features that help position the cells to the entrance of the migration channels, increasing the yield of the devices and minimizing the distance the cells travel before entering the migration channels. Similar to other devices for probing spontaneous cancer cell migration, the devices employed in this study rely on self-generated gradients of epidermal growth factor (EGF). These gradients are the result of a balance between cancer cells' EGF uptake in the loading channel and the restricted EGF diffusion through the migration channels from the fresh media in the external chamber, as described before in detail^11,17–19^.

Glutamine is the most abundant amino acid in the body (at around 0.6 mM in circulation). Glutamine is essential in the growth and division of cancer cells^20^, energy production^21,22^, lipid synthesis under hypoxic conditions^23^, ribonucleotide synthesis^24^, and protects the cells under oxidative stress^25^. Unlike prokaryotic cells, which have receptors for glutamine and other amino acids and employ them for directional migration towards energetic substrates^26^, no glutamine receptors have been found in mammalian cells. Instead, glutamine transporter molecules facilitate the entrance of glutamine inside epithelial cells and transport glutamine across the mitochondria membranes^27^. These transporters are not known to play any signaling role that could explain the role of glutamine in epithelial cell orientation.

When evaluating the role of glutamine in cell orientation, a major confounding factor can be cell proliferation, for which glutamine dependence is well known. This situation fundamentally limits the utility of current cell migration assays in studying the role of glutamine in orientation. The ubiquitous transwell migration assay evaluates migration indirectly, and a major confounding factor is the proliferation of the cells on both sides of the membranes. While epithelial cells could double their numbers in the ~ 24 h duration of the assay, and the process also dependes glutamine^28–30^, it is not possible to decouple the orientation during migration and proliferation. Thus, no rigorous determination of the effect of glutamine on epithelial cell migration is possible using the transwell assay.

Similarly, the traditional wound healing assays also lack utility when measuring the effect of glutamine on cell migration. The cells in the monolayer can increase their spreading area at the edge of the wound by movement and proliferation. Thus, one cannot distinguish between the effect of glutamine on cell migration and the effect of glutamine on cell proliferation when analyzing wound closure dynamics^9,10^.

A potential mechanistic insight into the role of glutamine in the directional migration of cancer cells may emerge from our observations that AKG bypasses the absence of glutamine. This suggests that a complete Krebs cycle is needed for the orientation of cancer cells. While the Krebs cycle takes place inside mitochondria, this result appears consistent with previous studies showing that the positioning of the mitochondria at the leading edge of moving cancer cells correlates with their directional migration abilities^31,32^ as well as with studies supporting a link between metabolism, mitochondria, and motility in cancer cells^33,34^. Moreover, our results challenge the assumed relationship between cellular metabolism and migration. Even though glutamine is often the major source of energy inside cancer cells, its absence alters cell orientation in the absence of major ATP level changes. Moreover, in non-cancerous epithelial cells, where ATP levels are altered in the absence of glucose, the changes in ATP levels do not alter cell orientation. Finally, while our measurements are focused on the trajectories of the first cells to enter each channel, we exclude the potential role of other signaling elements released by moving cells, e.g., extracellular vesicles, membrane debris, or secreted molecules, which could bias the migration of follower cells.

More studies are needed to determine if the glutamine-dependent cancer cell orientation we uncoveredance to cancer metastases in vivo. Candidate situations of low glutamine concentration and impaired cell orientation may exist in vivo at the center of tumors. There, the imbalance between high glutamine consumption by cancer cells and limited glutamine supply in the absence of proper vascularization^35^ may result in a local decrease in glutamine concentration. The white blood cells infiltrating the tumors, which are also known to consume large amounts of glutamine for their metabolism^36–38^, may further contribute to lower local glutamine levels. The new relationship between glutamine and cell orientation could suggest a new mechanism for trapping cancer cells inside tumors, delaying their spread. Emerging therapies targeting glutamine, recently reviewed elsewere^39^, may play further roles by facilitating the trapping of cancer cells and impairing their orientation during migration.

### Supplementary Information

Supplementary Information 1.

Supplementary Movie 1.

## Data Availability

The datasets analyzed during the current study are available from the corresponding author upon request.
