# Supplementary material for: Microfluidic devices for precise measurements of cell directionality reveal a role for glutamine during cell migration
Source: Sci Rep. 2023 Dec 27;13:23032. doi: 10.1038/s41598-023-49866-9 (PMC10754855; doi:10.1038/s41598-023-49866-9)
Supplement: Supplementary file 1 — Supplementary Information 1. [file 41598_2023_49866_MOESM1_ESM.pdf]

SUPPLEMENTARY MATERIAL

Microfluidic Devices for Precise Measurements of Cell Directionality Reveal a Role for Glutamine during Cell Migration

Nil Gural[a], Daniel Irimia[a,b,c,*]

a. Center for Engineering in Medicine and Surgery, Department of Surgery, Massachusetts General Hospital, Boston, MA.

b. Harvard Medical School, Boston, MA.

c. Shriners Hospitals for Children, Boston, MA.

* corresponding author: dirimia@mgh.harvard.edu

Supplemental Figure 1.

Supplemental Figure 2.

Supplemental Movie 1.

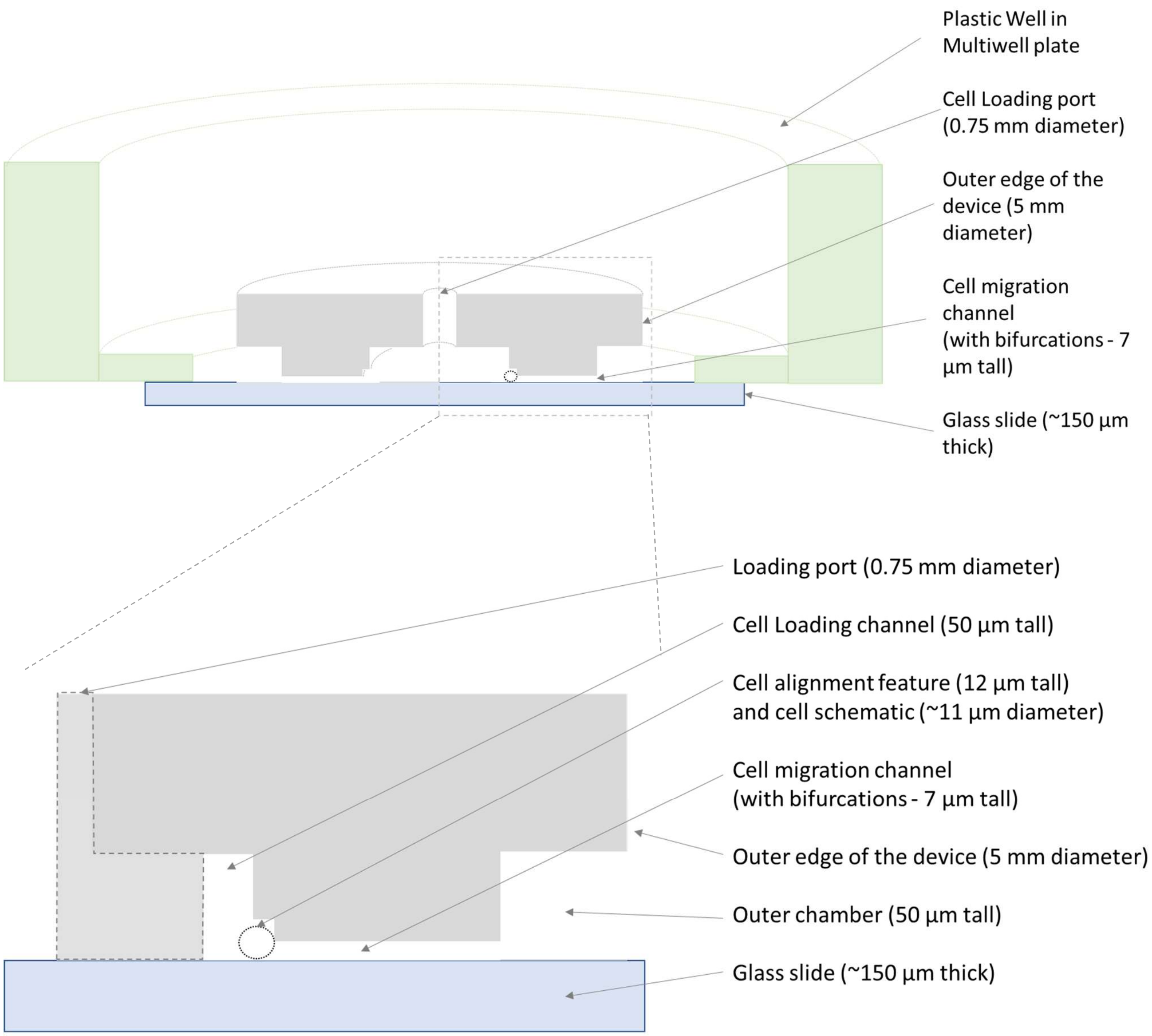

Supplemental Figure 1. Schematics of the device. **Top**: Cross section of the PDMS device inside a plastic well. The cell loading port is at the center of the circular PDMS device. The outlet is at the bottom between the PDMS and glass, at the end of the cell migration channels, throughout the circumference of the device. **Bottom:** Cross section of half of the PDMS piece bound to glass. The loading port and cell loading channels are out of the plane and are represented as a shade of gray and outlined by a dashed line. Cells loaded through the loading port and loading channels are pushed to the edge during loading, where they are mechanically trapped in the cell alignment feature, which has a height comparable to that of the suspended cells. The aspect ratio of the cell loading channel is such that the cells in a cell suspension distribute and cover the glass surface uniformly inside the loading channel. While individual alignment features trap cells at the entrance to each of the migration channels, the migrating cells will start moving approximately at the same time through the branching channels. The height of the migration channel is sufficient to accommodate the nucleus of the moving cells without mechanically squeezing it. After migrating through the channel, cells reach the outer chamber that is open to the edge of the device and the media in the well of the multi-well plate. An EGF gradient is established along the migration channel driven by the competition between the EGF uptake by the cells in the loading channel and the EGF diffusion from the outer chamber through the migration channels. The self-generation mechanism for EGF gradients in this type of microfluidic device has been described before in detail [reference 11].

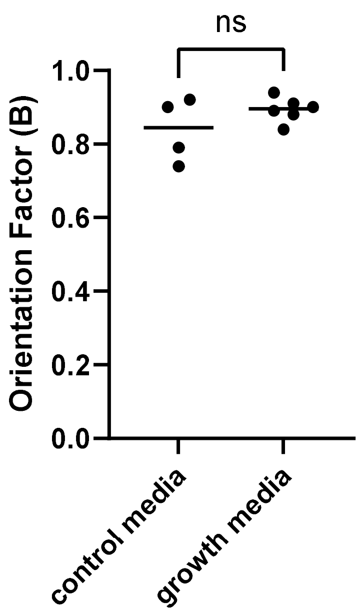

Supplemental Figure 2. Comparison of orientation of PC9 cell in growth media vs. control media. The difference between the conditions is not significant ($p>0.05$, $N >= 4$).

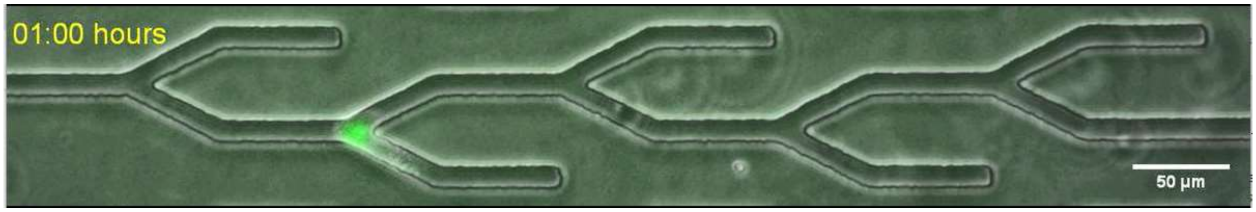

Supplemental movie 1. GFP-expressing PC9 cell passes on bifurcation and gets temporarily trapped in the second bifurcation. Scale bar is 50 µm. The duration of the movie is 4 hours.
